# Crowd of Oz: A Crowd-Powered Social Robotics System for Stress Management

**DOI:** 10.3390/s20020569

**Published:** 2020-01-20

**Authors:** Tahir Abbas, Vassilis-Javed Khan, Ujwal Gadiraju, Emilia Barakova, Panos Markopoulos

**Affiliations:** 1 Department of Industrial Design, Eindhoven University of Technology, 5600 MB Eindhoven, The Netherlands; v.j.khan@tue.nl (V.-J.K.); e.i.barakova@tue.nl (E.B.); p.markopoulos@tue.nl (P.M.); 2 Department of Software Engineering, Mirpur University of Science & Technology (MUST), Mirpur AJK 10250, Pakistan; 3 L3S Research Center, Leibniz Universität, 30167 Hannover, Germany; gadiraju@l3s.de

**Keywords:** social robotics, coaching, social conversation, crowdsourcing, human computation, real-time crowd-powered systems, stress

## Abstract

Coping with stress is crucial for a healthy lifestyle. In the past, a great deal of research has been conducted to use socially assistive robots as a therapy to alleviate stress and anxiety related problems. However, building a fully autonomous social robot which can deliver psycho-therapeutic solutions is a very challenging endeavor due to limitations in artificial intelligence (AI). To overcome AI’s limitations, researchers have previously introduced crowdsourcing-based teleoperation methods, which summon the crowd’s input to control a robot’s functions. However, in the context of robotics, such methods have only been used to support the object manipulation, navigational, and training tasks. It is not yet known how to leverage real-time crowdsourcing (RTC) to process complex therapeutic conversational tasks for social robotics. To fill this gap, we developed Crowd of Oz (CoZ), an open-source system that allows Softbank’s Pepper robot to support such conversational tasks. To demonstrate the potential implications of this crowd-powered approach, we investigated how effectively, crowd workers recruited in real-time can teleoperate the robot’s speech, in situations when the robot needs to act as a life coach. We systematically varied the number of workers who simultaneously handle the speech of the robot (N = 1, 2, 4, 8) and investigated the concomitant effects for enabling RTC for social robotics. Additionally, we present Pavilion, a novel and open-source algorithm for managing the workers’ queue so that a required number of workers are engaged or waiting. Based on our findings, we discuss salient parameters that such crowd-powered systems must adhere to, so as to enhance their performance in response latency and dialogue quality.

## 1. Introduction

Psychological stress is an extent to which persons observe that their demands exceed their ability to manage. Coping with stress is crucial for a healthy lifestyle. Prolonged and high level of stress in humans can affect several physiological and psychological functions including change in heart rate and heart rate variability, muscle tension in the neck, hormonal changes [1] and cause negative feelings. Stress can also affect memory processes [2] and can impact the ability to learn and remember [2].

University students are more susceptible to develop stress and anxiety related problems due to highly technical and emotional demands of their studies. Sometimes, psychological suffering is so severe that it could lead them to suicidal ideation [3,4] or dropout from university. The most common stressors that could persuade mental illness and emotional distress include but are not limited to academic overload (e.g., assignments, exams), sleeping problems, transition to a new environment, not being able to enjoy day-to-day activities, loneliness, being away from home, lack of self-efficacy and optimism, and not being able to make new friends [5,6,7,8,9].

In the past, a great deal of research has been conducted to leverage socially assistive robots as a therapy to alleviate stress and anxiety related problems among children [10], older adults [11], and teens [12,13]. However, building a fully autonomous social agent which can deliver psycho-therapeutic solutions is a very challenging endeavor which requires emotional intelligence, affect analysis, computational psychology, automated speech recognition, natural language processing (NLP), prediction and planning, and advanced computer vision techniques to automatically recognize non-verbal cues such as gestures, facial expression, and objects [14]. Consider, for example, holding a supportive discussion with someone who feels stressed—we will refer to this act as ‘stress mitigation’. For a stress mitigation task, the robot, acting as a coach, should be able to establish a sympathetic relationship with the user. Namely the robot must: help the user to understand his/her feelings through asking open questions; reflect and validate the client’s problems; and exhibit some action skills by stating opinions, facts and solutions based on the user’s situation. This requires robots to go beyond the current AI methods and acquire specialized skills in affect analysis and computational psychology. Therefore, fully automated, open-ended discussion with social robots remains elusive.

Crowdsourcing seems to be viable solution that can overcome the aforementioned problems. In the past, researchers have investigated the use of crowdsourcing for delivering positive psychological interventions to people who are stressed [15]. This concept is known as *affective crowdsourcing* [16]. For instance, Emotional Bag Check [17] is an emotional support tool that rely on a cohort of volunteers and trained people to give therapeutic support to students. Panoply is a crowd-powered system that leverages the wisdom of crowd to provide on-demand emotional support to people anytime and across the globe though cognitive reappraisal technique. Nevertheless, these emotional support tools take enormous amount of time to give empathetic responses to stressed peoples. For instance, Panoply takes on average 9 min to 2 h to receive a first response from crowd workers and other registered users, which is not suitable for a two-way live synchronous conversation.

This paper investigates rapid crowdsourcing techniques to summon a synchronous group of workers in real time to provide therapeutic support through two-way live synchronous conversation. It was made possible thanks to the development of new methods that help to reduce the latency of the crowd’s input from hours to seconds [18]. Such systems have introduced the notion of real-time, synchronous crowdsourcing (RTC) where the crowd engages synchronously in processes that are characterized by real-time constraints. Recently, RTC based teleoperation methods have emerged as a plausible alternative for teleoperating robots and for supporting machine learning [19]. For instance, Legion [20] crowdsourced a toy robot’s locomotion to a real-time synchronous crowd. Another example is EURECA [21] where online crowds assisted robots to identify unknown objects in a scene in real-time. Nevertheless, prior research has focused only on enabling RTC to support the robot in navigation and manipulation tasks. However, to this point it has not been attempted to apply RTC to enable two-way live communication for social robots to handle complex therapeutic tasks.

Furthermore, previous emotional support tools rely on a text based medium for exchanging messages which is not perceived as enjoyable and interactive as compared to an embodied conversational agent. For example, Berry et al. [22] compared an animated embodied agent (GRETA), with a voice-based agent and text only agent. Their participants rated GRETA as more likeable and helpful as compared to the text-based and voice-based agent. In another study [23], Powers et al. compared a collocated robot with a remote projected robot and an agent on the monitor screen. Their results show that the collocated robot was more engaging, more helpful, gave better advice and was more effective communicator than the projected robot and animated agent. Hence, these studies motive us to use the embodied conversational agent (Softbank’s Pepper Robot) as a life coach to deliver a psycho-therapeutic solution.

This paper presents the first attempt in applying RTC to facilitate an embodied conversational agent to provide emotional support to people who are stressed. This presents several challenges from a crowdsourcing perspective, pertaining to sustaining the context of the conversation and ensuring the speed and fluency of the robot’s behaviors. We have developed an open-source system called Crowd of Oz (CoZ) to enable crowd-powered social robotics for therapeutic tasks. Firstly, CoZ broadcasts a live audio–video (AV) stream of an interlocutor, to anonymous workers online who can then communicate with a stressed person through the embodied conversational agent in real time. In this version of CoZ, we only focused on controlling the speech of the robot and gestures were controlled by Pepper’s built-in animated speech API (application programming interface), which automatically produces appropriate gestures based on the text input. Secondly, CoZ features a crowd user interface (UI) that supports them in promptly typing utterances based on the context of the conversation. Finally, CoZ supports a novel workflow to handle the asynchronous arrival and departure of workers during the task.

To demonstrate the feasibility of this approach, we investigated how effectively a crowd of workers recruited in real-time, can act as a coach by teleoperating the Pepper robot [24] in a stress mitigation task. To investigate this, we invited a professional actress who played the role of a stressed Master’s student and improvised dialogues with the Pepper robot (supported by CoZ) in different sessions. For each session, we systematically varied the number of workers who simultaneously controlled the robot’s speech. Our results indicate that crowd input was both meaningful and fast enough to hold a sensible conversation. Finally, we asked two professional psychologists to assess how effectively the crowd was able to provide life coaching to the user. Their assessment indicates that the crowds’ performance was satisfactory. Based on the series of experiments and the psychologists’ feedback, we propose guidelines for enabling RTC to support complex conversational tasks for social robotics.

Contributions: We contribute to the body of literature a proof of concept demonstration of how RTC can enable a social robot to handle a social conversation. Specifically, we:

1. present CoZ, a system designed to crowdsource the teleoperation of a social robot for conversational tasks. CoZ does that by: (a) live streaming the robot’s AV feed to workers, thereby enhancing their contextual and social awareness, (b) managing workers’ asynchronous arrival and departure during the conversational task, (c) supporting workers’ task performance through its UI. Our entire code base is open source and is available on Github (https://github.com/tahir80/Crowd_of_Oz).
2. evaluate the trade-off between response latency and dialogue quality by systematically varying the number of workers. We release our data set (https://doi.org/10.6084/m9.figshare.9878438.v1) containing all dialogues between CoZ and the actress to promote further research.
3. provide RTC-specific guidelines for social robots operating in complex life-coaching tasks.

## 2. Related Work

### 2.1. Remote Teleoperation

Remote teleoperation will remain an important subject of research due to limitations in the current levels of autonomy offered by artificial intelligence [25,26]. Teleoperating a humanoid or social robot can be formally defined as [27]:

⋯ the process through which a human directs remote sensors and manipulators using a communication channel subject to latency and bandwidth limitations in such a way that robot behaviors are physically, emotionally, socially, and task appropriate.

Several methods have been used to achieve realistic and meaningful conversations with robots. Foremost, the Wizard of Oz (WoZ) method is widely applied in experimental settings to test hypothesis without the need of extensive programming, and has been used in education [28], and elderly care centers [29] and in public spaces [30]. In applications that specialised knowledge and expertise is needed, previously developed interactive scripts with specific purpose are used and supported by, for instance a physical game, to streamline the interaction [31]. This method can only facilitate a restricted range of conversations, and is not sustainable for longer term interactions. To improve the interactivity, the predefined scenarios can be connected to an interface that allows adding in real-time of new speech utterances if an unexpected situation occurs [32]. In addition, the direction of the interaction, and thus the conversation can be done by an operator. A third group of applications attempt to use natural speech recognition and generation, however these attempts are used with a limited success in real life applications, since they can only achieve a limited understanding of the context of the interaction [33].

In this study, we used limited robot autonomy (Pepper’s animated speech) and crowd workers were allowed to manually control the conversation of the robot through a web interface. Furthermore, we permitted cooperative control where multiple workers were invoked to control the speech of a single robot simultaneously.

### 2.2. Crowdsourcing

Crowdsourcing is an emergent approach to collaborative work that provides easy access to the skills and talent of large number of individuals spread geographically, to solve tasks that would otherwise be difficult or costly to solve when using traditional approaches [34]. The typical form of crowdsourcing is known as microtask crowdsourcing, which allows people to globally distribute short and simple tasks that do not require any particular expertise to various online workers (also known as crowd-workers). Workers then solve those human intelligence tasks. Examples of such tasks include: image labeling [35]; writing and editing [36]; protein folding [37]; and generating creative contents [38], among others.

### 2.3. Real-Time Crowd-Powered Systems

Real-time crowd-powered systems use recruiting, rewarding and user interface techniques to reduce latency of crowd input from hours to few seconds [18]. In traditional crowdsourcing systems, tasks are often completed in batches and take hours or days making the collaboration between requesters and workers essentially asynchronous. In recent years, researchers have developed methods to recruit workers on demand from crowdsourcing platforms and created techniques to reduce latency. Such methods enable real-time crowdsourcing (RTC) [39] and the resulting systems that build on top of the RTC concept are known as real-time crowd-powered systems. VizWiz [40] is one of the earliest real-time crowd-powered system which helps blind users in answering visual questions about their surroundings by sending object’s photos and audio questions from their phones to crowd workers. Chorus [41] is a text-based chatbot that assists end-users with information retrieval tasks by conversing with online synchronous group of workers. To automate conversation, Evorus [42] builds on Chorus by employing both machine learning and human computation to enable a group of crowd-workers to collaborate with chatbots. Although effective, this approach is constrained by the availability of suitable domain specific chatbots and to purely text-based communication. Chorus: view [43] extends VizWiz by providing real time video stream and a recorded audio question to workers in addition to text based chat to further improve the capability of VizWiz in answering general questions to visually-impaired users. InstructableCrowd [44] is a conversational system which allows end users to verbally communicate their problems regarding IF-THEN rules to a synchronous group of workers. In return, crowd workers assist end-users by collectively programming their sensors and actuators on their mobile phones. Finally, CrowdBoard [45] allows collocated ideators to develop ideas on a digital whiteboard with the help of synchronous remote crowd workers sharing the same digital whiteboard. We have highlighted some key differences between CoZ and other related systems from real-time crowd-powered systems domain in Table 1.

### 2.4. Crowd or Web Robotics

RTC has been used in the past as a training and teleoperation method for robotics. For example, Legion [20] allows end-users to crowdsource the real-time synchronous control of the locomotion of a toy robot through keyboard commands, while observing a real-time video feed. Likewise, CrowdDrone [46] replaces a human operator with a cohort of workers to control unmanned aerial vehicles in an unknown environment and supports novel mediation strategies for aggregating commands by crowd-workers. EURECA (Enhanced Understanding of Real Environments via Crowd Assistance) [21] helped robots understand unknown objects from a scene in real-time by leveraging online crowds of human contributors. Robot management system (RMS) [47] is an open-source web-based framework which allows researchers to outsource navigation and manipulation tasks of the PR2 robot to anonymous and diverse users from across multiple demographics. In another similar work, Crick et al. [48] leveraged crowdsourcing to teach the robot through demonstrations.

The case of controlling socially assisted robots that we examine here has several similarities but also crucial differences with the aforementioned applications. The similarities start and finish in the real-time nature of the task. The crucial differences are that in contrast to a task that has a specific answer or requires choosing among a small set of specific answers (e.g., which direction to move towards), holding a conversation is an open task with many options that unfolds over time. Furthermore, beyond a low latency of responses the robot must exhibit empathy, ask relevant questions and provide meaningful suggestions in the light of what has been said throughout the course of the dialogue exchange. We address all of these differences in our investigation of CoZ in this paper. We have highlighted some key differences between CoZ and other related systems from crowd or web robotics domain in Table 2.

### 2.5. Social Robotics and Stress

In the past, a great deal of research has been conducted to use socially assistive robots as a therapy to alleviate stress and anxiety related problems among children [10], older adults [11], and teens [12,13]. In a recent study [10], it was reported that children who interacted with the robot showed an increase in positive mood as compared to two other conditions (where the robot was turned off, or was waiting quietly). More recent review of social robots for the well-being of older adults revealed that social robots can reduce loneliness and stress and can enhance engagement [11]. Furthermore, teens were also found to show strong engagement and expressions of empathy even with a low-fidelity prototype [49], and also having participated as a designer of a robot that could overcome their stress [12].

### 2.6. Latency in Crowdsourced Tasks

In addition to task latency, which is domain specific, key to enabling RTC is to reduce the recruitment and arrival latency of workers. There have been various strategies proposed for doing so. For example, quikTurkit [40] reduces worker’s arrival latency by recruiting workers in advance. It posts in batches many more tasks than are actually required to keep tasks at the top of MTurk’s list of tasks for crowd-workers to choose from. While in a waiting pool, workers are kept engaged by performing old tasks posted by the requester. It also posts tasks with slightly different titles and payments to keep tasks at the top of MTurk’s list.

Rather than engaging workers with old tasks, an alternative strategy is the Retainer [18] where workers are paid fixed fee for waiting and a small reward (3 cents) for quickly responding to alerts. Nevertheless, waiting in a retainer for a fixed duration and then responding quickly is not appreciated by workers [50]. Furthermore, if payment for waiting is lower than the US minimum hourly wage, workers can perceive it as unethical and unjust.

More recently, the Ignition recruitment process [50] proposes a combination of both on-demand recruitment and waiting pool mechanisms to reduce recruitment latency. Ignition recruits more workers than needed, keeping extra workers in a retainer pool. Our recruitment algorithm Pavilion is inspired by Ignition but differs in the way it handles a worker’s turnover conditions during the execution of task (e.g., when workers leave the task either by submitting their work or returning the human intelligence task (HIT)) and in that it hires extra workers parsimoniously only when needed.

## 3. Crowd of Oz System

**Figure 1:**
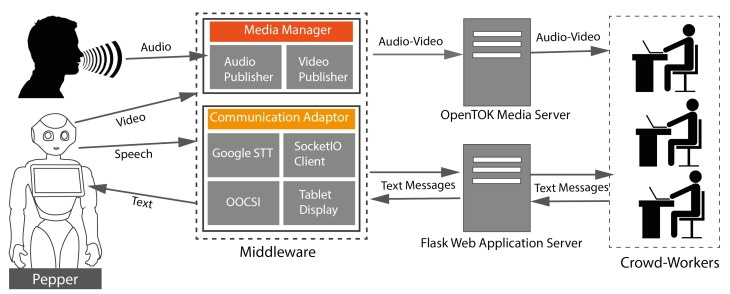
High-level system architecture of Crowd of Oz.

CoZ conceptually consists of four major components: the Pepper robot, the middleware, the Flask web app, and the crowd interfaces (see Figure 1). Before delving into the details of the system, we present a high level illustration of CoZ. When a user speaks to the robot using the microphone, the speech recognition script running on the middleware detects the user’s voice and converts it to text. At the same time, the middleware sends a signal to the application server to activate the controls on the crowd interface as we allow workers to send a message only after a user says something. When the transcribed text is available, it is also sent through the same channels to the server. On the server side, the Flask app receives the message, displays it on the crowd interface for workers and displays it to the Pepper robot’s tablet for the user.

When a crowd-worker wants to reply, instead of typing she can use the speech to text service. After her spoken message is automatically transcribed, she can click the button “Send”. On the server side, the Flask app receives the message(s), selects a message based on the first-response strategy (for this version of CoZ, we implement first-come-first-serve strategy to speak out the first available message on the Pepper robot), displays that message to the Pepper robot’s tablet through middleware and rewards the worker with a bonus. Most importantly, the Flask app also sends a message to the Pepper robot through middleware. It then uses Pepper robot’s text-to-speech (TTS) API to execute animated speech—Pepper’s animated speech includes gestures based on the text input.

### 3.1. Pepper Robot

We used Softbank’s semi-humanoid Pepper robot (https://www.softbankrobotics.com/us/pepper). Pepper’s execution environment is a customized version of Linux called Gentoo. The Pepper robot is equipped with a camera, microphone, speakers, and a tablet for providing textual and graphical output to the user. Pepper can exhibit some social behavior, e.g., gestures, head movements, and adjust its head pose to track users.

### 3.2. Middleware

The middleware runs on a Windows PC and acts as a bridge between the application server and Pepper. All audio–video (AV) and text communication are handled by the middleware. The middleware runs two main components: the media manager and the communication adaptor.

#### 3.2.1. Media Manager

The media manager includes audio and video publishers. The audio publisher implements OpenTOK API (https://tokbox.com/) and broadcasts audio of a user from the collar microphone. The video manager also implements OpenTOK API and broadcasts video feed from the Pepper’s front camera to the crowd interfaces. For real time AV transmission, we use the WebRTC protocol (https://webrtc.org/). WebRTC is a free, open source technology that enables real-time AV and text communication to modern browsers and mobile apps. In this system, we use OpenTOK APIs which have built-in support for WebRTC technology.

#### 3.2.2. Communication Adaptor

The communication adaptor runs four major programs: Speech recognition, the OOCSI sender/receiver clients (https://github.com/iddi/oocsi-python), the Flask based SocketIO client (https://flask-socketio.readthedocs.io/en/latest/) and a script to display messages on the Pepper’s tablet. The speech recognition module converts speech into text and forwards it to the OOCSI client. OOCSI is a prototyping middleware for designing distributed products and supports connecting multiple heterogeneous client programs through the WebSocket protocol. In this project, we used OOCSI clients to connect the middleware with the Pepper robot through WebSocket for exchanging text messages. The SocketIO client connects the middleware with the application server. It handles all external messages with application server through a WebSocket secure connection.

### 3.3. Flask Web App

We developed the server application using Flask (http://flask.palletsprojects.com/en/1.1.x/), a Python-based web framework, while for the client, we used Bootstrap, JQuery, and CSS. We built the application modular using Flask blueprints (https://flask.palletsprojects.com/en/1.1.x/blueprints/). Each blueprint is considered as an independent application serving its own functionality. We have three major blueprints in our application: (1) admin panel; (2) authorization; (3) crowd control. Crowd control is the crux of the application and it includes the necessary logic to handle crowd interfaces and manage crowd workers throughout the task.

### 3.4. Crowd Interfaces

**Figure 2:**
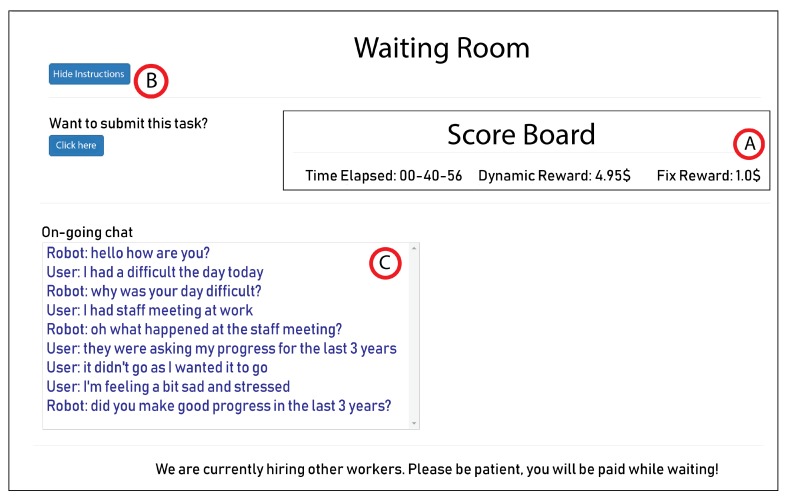
Waiting page where workers are kept to ensure availability.

First, the administrator creates a new project and a new HIT on the Amazon Mechanical Turk (hereafter: MTurk) through the admin control panel. Creating a HIT requires several attributes: title, description, fixed price, qualification requirements, maximum number of workers etc. After that, the HIT is posted to MTurk; this is transparent to the admin as it is done through CoZ’s interface. Once the HIT is posted on MTurk, crowd workers can read the instructions and accept it. After accepting, they are directed to the waiting page (Figure 2) where they remain until sufficient number of workers have been recruited. They can also read the detailed instructions (point B on Figure 2). If the session has already been initiated, workers can see the on-going chat between the user and robot on the waiting page (point C), thereby becoming aware of the context of the conversation. Workers are financially compensated according to average US minimum wage ($7.25/h) while waiting (point A).

**Figure 3:**
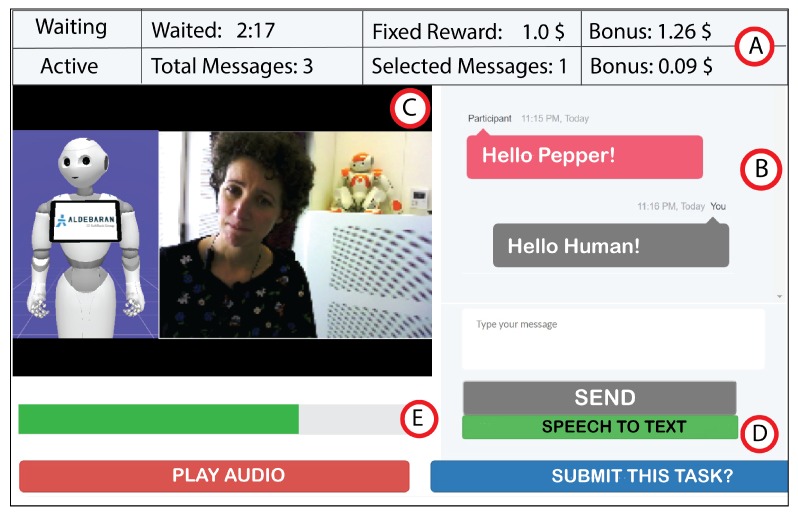
Crowd worker’s main task page. It also shows an actress interacting with the CoZ from one of the sessions.

The main page contains a task ribbon at the top of the page (cf. Figure 3) showing a worker’s task-related information including accumulated bonuses based on their contributions (point A). Furthermore, it displays a chat history (point B) between user (red) and workers (grey). On the left side, we display the real-time view of the robot’s 3D model, an AV feed (point C) and a button for using speech-to-text based on IBM’s Watson API (point D). We encourage workers to use this speech-to-text functionality to reduce latency. To nudge workers for swift responses, we implemented a progress bar (point E). When the user stops speaking, a progress bar sets off for a maximum of 7 s with additional 2 s to determine the pause. This signaled workers to compose and submit their message during these 9 s. Furthermore, they could also see messages from other workers on the chat box interface to avoid asking same questions and to maintain the continuity and coherence of the discussion.

## 4. Pavilion Algorithm

As mentioned earlier, our open-source (Our implementation of Pavilion and installation details are available at: https://github.com/tahir80/Pavilion) Pavilion algorithm extends the one introduced with Ignition, with new features to handle turnover conditions (i.e., returning the task) both in the waiting and active queue. Pavilion initially hires a fixed number of workers (based on the total number of active and waiting workers required) and then prudently hires more workers if needed. Hence, the total number of workers that we need to hire from MTurk initially are equal to:

(1) $$H_{Mturk}\;=\;W_{max}+A_{max}$$

$A_{max}$ denotes the maximum number of active workers who can work on the actual task simultaneously. $W_{max}$ denotes the maximum number of target workers who can wait in the waiting queue simultaneously. Similarly, $W_{min}$ represents the minimum number of workers allowed in the waiting while $A_{min}$ represents the minimum number of active workers required to initiate the dialogue. In the experiment we report below, we wanted to initiate the conversation when $A_{min}$ workers were hired, so we set $A_{max}$ = $A_{min}$. Nevertheless, this condition can be changed.

The total number of workers in the waiting queue ($W_{max}$) will always be greater than the total number of workers in the active queue ($A_{max}$) by 1:

(2) $$W_{max}\;=\;A_{max}+1.$$

Our rationale behind choosing one extra worker for the waiting queue was to keep at least one worker in the waiting queue when the dialogue was initiated. Thus, the minimum number of waiting workers ($W_{min}$) is always equal to 1. Furthermore, we aim to reduce the chance that the waiting queue will be empty.

### 4.1. How It Works

1. Initially, Pavilion keeps adding workers in the waiting queue until the current count of waiting workers becomes equal to $A_{min}$. At that point, when a new worker joins the session, all previous workers who were waiting are transferred to the active queue to initiate the conversation (except the one who has most recently joined the session who stays in the waiting queue). For the task to start we expect at least one worker to be in the waiting queue $W_{min}\;=\;1$.
2. On the background, Pavilion continues adding workers in the waiting queue during the execution of the task until the maximum condition for the waiting queue is reached: $W_{max}\;=\;A_{max}+1$.
3. When a worker leaves the active queue (from the actual conversation task) either by submitting the task or returning it. (There is a difference between submitting and returning a HIT. In submitting, a worker leaves the task by actually submitting the HIT to MTurk for reviewing and rewarding. While in returning, a worker leaves the task but is not interested in the monetary reward and the returned HIT is available for other workers on MTurk.), Pavilion immediately pushes one worker from the waiting queue to the active queue to keep the target number of workers fixed. If the task is actually submitted by the worker, then Pavilion also posts one extra HIT (with one assignment) to fulfil the deficiency in the waiting queue.
4. When a worker leaves the waiting queue by submitting the task, Pavilion hires a new worker. Nevertheless, when a worker leaves the waiting queue by returning the HIT, Pavilion does nothing because the returned HIT is immediately available to new workers on MTurk.
5. In the worst case, when the waiting queue only contains one worker or none and a worker leaves from the active queue, then Pavilion waits until another worker(s) joins the session and then it moves a worker(s) from the waiting to the active queue until the following condition is false:
  (3) $$\left(W_{C}-1\right)>\left(W_{min}-1\right)\wedge \left(A_{C}+1\right)<=A_{max}$$
  where $W_{C}$ and $A_{C}$ represents the current number of waiting and active workers respectively.

### 4.2. Differences between Pavilion and Ignition

Pavilion is designed for supporting short-term tasks that require a specified number of workers throughout a session. Ignition on the other hand is intended for long-term jobs and can initiate the dialogue as long as there is one worker in the session.

Ignition only manages the active queue when the total number of active workers falls below a minimum threshold, while Pavilion manages both the waiting and active queues and posts new HITs during the task to fulfil the deficiency of workers in both queues.

Ignition does not allow workers to leave the task throughout the session; workers have to commit a lot of time and wait until Ignition auto-submits their HITs. In contrast, Pavilion adopts a more flexible approach by letting workers leave the task by actually submitting their HITs both from the waiting and the active queues when they do not want to continue, e.g., due to fatigue or boredom or because they are satisfied with the bonus they earned. We also aimed to safeguard for the possibility that workers would get upset or anxious from the discussion, for which an easy exit with compensation was deemed necessary and appropriate.

## 5. Materials and Methods

**Figure 4:**
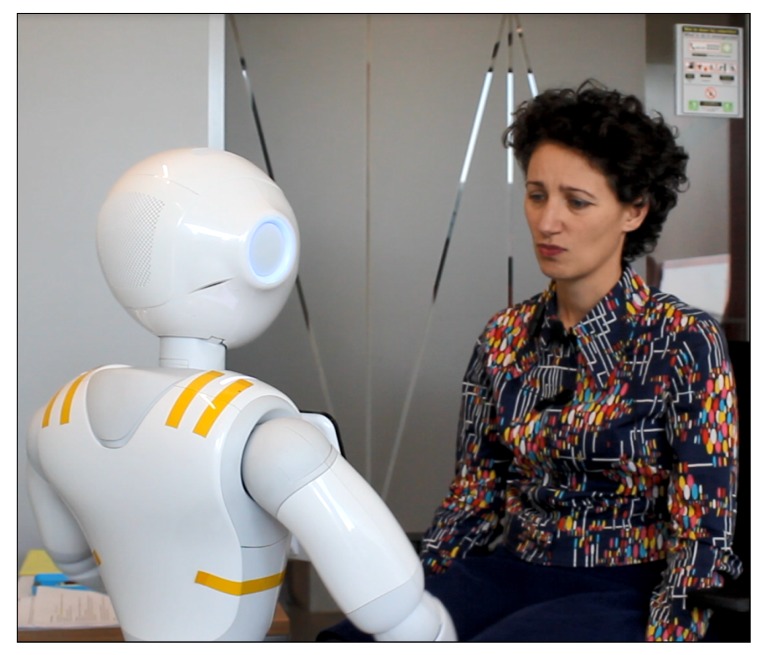
An actress interacting with a CoZ powered Pepper robot during a session.

We conducted experiments to investigate how varying the number of workers (N=1,2,4,8) affects the quality of conversation and the real-time performance of CoZ. Workers were asked to type-in what the robot would say in a conversation with a student suffering from study-related stress. Our focus was on the effectiveness of the coaching and the human computation aspects of CoZ, so the student was enacted by a professional actress (Figure 4). We opted for this approach for methodological, ethical, and practical reasons. First, given that this is the very first study to investigate leveraging crowdsourcing to tackle psychological distress, our focus was the performance of the crowd in relation to the system we build (CoZ). Therefore, we wanted to have as similar as possible interactions from the user’s point of view but at the same time engage the crowd workers in a realistic interaction. Second, we took care to recruit an actress that specialize in improvisational theatre precisely because we wanted to offer crowd workers a realistic interaction. Having our actress as a single user also offered us her perspective in terms of her experience of the different conditions of our study, when we debriefed her. Thus, the above two reasons address the methodological dimension of our choice.

Third, given that the topic is a sensitive one and given that we did not precisely know how the crowd will perform, we did not want to put in an even more difficult situation actually stressed students. In other words, we were very careful in not potentially causing distress or harming any participant in our study. Thus, this was the ethical dimension of our choice.

Finally, there is also a practical dimension of our choice, namely efficiently executing the study since having the actress as a single user could allow us to execute several sessions throughout a day.

### 5.1. Task for Crowd Workers

On MTurk, we described the task as:

You are asked to act as a teleoperator of a robot and chat with a university master student who is experiencing stress due to study burden and not being able to keep work-life balance. Your task is to empathize with the student through conversing and try to find out why the student is stressed by asking open questions. Only after having a good understanding of the context and only when you have asked several open questions think about politely suggesting solutions to student to get out of this stressful situation.

Furthermore, crowd workers did not receive any formal training about cognitive therapy, but they were given detailed instructions about the nature of the task and how to use the user interface accurately.

### 5.2. Participants

We recruited workers on MTurk and restricted the study to US workers, but had one session with Indian workers, with over 98% approval rating and 1K HITs approved. We made sure to compensate each worker with at least the average US hourly wage ($7.25). Specifically, we had a $1.0 fixed reward, plus the following bonuses: $7.25/h while waiting in the queue; 2 cents for providing a response; and an extra 3 cents if their response was selected by our algorithm. We recruited a total of 245 workers. Of these, 102 workers stayed till the end of the task, while the remaining workers left during the course of task completion. The total cost of the study was $335.

### 5.3. Procedure

We had four conditions for recruiting workers to input the utterances spoken by our robot: (C1) single worker; (C2) a pair of workers; (C3) a group of four; (C4) a group of eight. Our reason for using this geometric sequence (doubling the number of workers in different conditions) was that in the absence of prior knowledge as to the number of workers needed, we wanted to ensure sufficient variation between the number of workers recruited in the different conditions. We randomized all conditions and each condition was executed five times (sessions) with our actress. This results in total of 20 interactions of our actress with our CoZ-enabled robot. We conducted the sessions across two different days and at different times of the day to ensure that unique workers contributed in each session, and that differences due to tiredness or time would balance out across conditions. The average dialogue duration was 11.27 min (SD = 1.74). Before initiating the experiments, we discussed the study’s purpose and the role that was needed to be played with the actress in detail. We also informed her about the live AV broadcast to the anonymous workers. The actress did not prepare or follow a predefined script and she was allowed to control the flow of discussion based on the crowd workers’ responses.

### 5.4. Measures

(I) Response latency: the time (in seconds) between our actress’ utterance and the first response received from the crowd for each dialogue in all conditions. (II) Dialogue quality: we used appropriateness as a measure for dialogue quality [51]. Appropriateness is a measure that computes a score at each exchange level or dialogue turn rather than for the entire dialogue. Each dialogue turn is given a single label (e.g., appropriate/inappropriate) which translates to a numeric value. We coded each dialogue turn (robot utterance) as “appropriate” when the robot responds appropriately to an utterance or request (with score: +2). On the other hand, we coded each dialogue turn as “inappropriate” which was off-topic, irrelevant, or confusing (with score: −1). Finally, we penalize those responses when the workers fail to understand the actress’ utterance and requests to repeat it. We did this because workers can ’listen’ to the user well and can also ’see’ the text-based response on their chat window screen unlike AI which could fail for many reasons (e.g., due to noise in the background). Hence, it was unlikely that they misunderstood the user except in cases when they could not respond for unforeseen reasons. Therefore, we treated this as a ’separate’ measure to penalize the dialogue (with score: −0.5) when this occurred. (III) Assessing the quality of robot utterances through linguistic inquiry and word count (LIWC): LIWC is a simple text analysis program that reads the given text and counts the percentage of words that reflect emotionality, social relationships, thinking style etc. [52]. Currently, LIWC searches for words across more than 80 built-in categories. We choose the following variables: emotionality (positive (posemo) and negative emotions (negemo)), word count (WC), words per sentences (WPSs), and difficult words (words comprise of more than six letters (Sixltr)). (IV) Average waiting time for eliciting multiple responses: The waiting time before a sufficient number of responses to choose from has been received. (V) Effect of progress bar and speech-to-text (STT) on latency: Since we implemented a progress bar in the crowd interface (with a maximum elapsing time of 9 s) and the STT service, we were interested in understanding the effect of these on the overall response latency. (VI) Experts’ evaluation and professional feedback: We consulted two professional clinical psychologists, who we recruited from Fiverr (https://www.fiverr.com/), and were experts in depression and anxiety related disorders. The purpose was to evaluate workers’ overall life coaching skills through the eyes of specialists. In this way, we can ensure the validity of ratings received from therapists who were well-versed with cognitive behavioural therapy (CBT) and motivational interviewing (MI) techniques. We also want to assess the qualitative aspects of life coaching delivered by crowd workers. For instance, what were the strengths and weaknesses of responses provided by workers? Did the workers unwittingly follow some good medical practices based on CBT or MI? Finally, based on the qualitative feedback, we want to establish some guidelines for training workers in future for these therapeutic tasks. The psychologists rated the quality of the dialogues on a seven-point Likert scale (1: highly unprofessional to 7: highly professional). Furthermore, they also provided detailed feedback on four randomly selected dialogues—one from each condition.

## 6. Results

### 6.1. Summary of Crowd Responses

As we mentioned before, the actress did not use any script or predefined contents to control the flow of the dialogue, though by reviewing the dialogues, we noticed some repetitively occurring problems that actress mentioned. In Table 3, we have summarized those problems or situations and corresponding crowd responses. In addition to crowd workers’ empathetic responses to actress’ worries, they provided some practical solutions to resolve them. Table 4 shows the summary of solutions provided by crowd workers.

### 6.2. Response Latency

**Figure 5:**
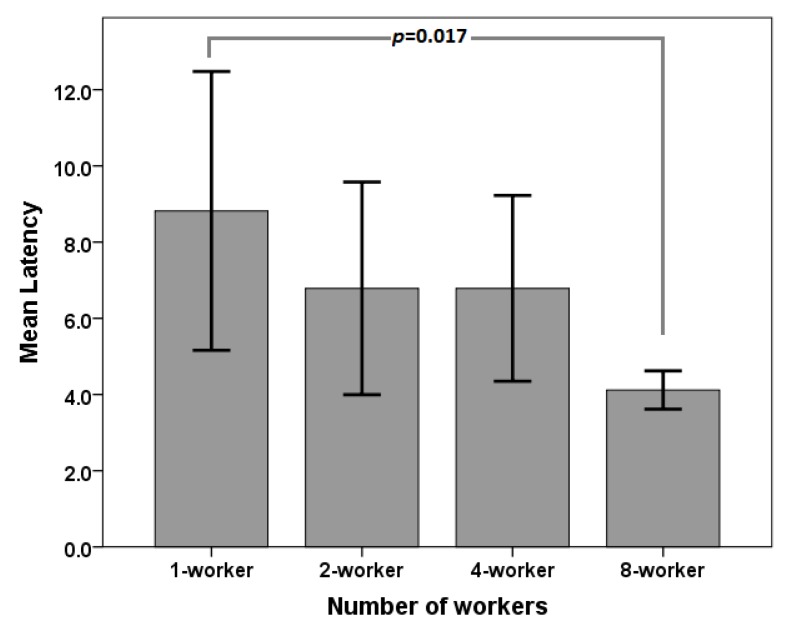
Mean latency: significant difference in the average response time between one-crowd and eight-crowd conditions.

A one-way analysis of variance (ANOVA) showed a significant difference in mean latency scores between the four conditions; F(3,16)=4.184, p=0.023. Post-hoc Bonferroni comparisons indicated that this difference in latency was significant (p=0.017) between the one- and eight-worker conditions (cf. Figure 5 and Table 5). We did not find any difference between the other conditions.

Since we observed a linear drop in the latency in relation to the number of workers (Figure 5), we investigated whether the number of workers can predict the latency. A linear regression model confirmed that the number of workers in the active queue were able to predict the variance in response latency scores. The linear regression model explained 39.3% (medium) of the overall variance in the mean latency scores ($R^{2}$ = 0.393), which was found to significantly predict the outcome, F(1,18)=11.66, p=0.003, effect size (*d*)=0.647. The linear equation was:

(4) Latency=8.84−0.589(number_of_workers)

Though we experience a reduction in latency of about 0.6 (0.589) seconds per new worker added, we expect that latency should level out after a certain number of workers have been added. Hence, investing more money would be superfluous beyond such a limit. Future studies should determine this limit.

### 6.3. Dialogue Quality

**Figure 6:**
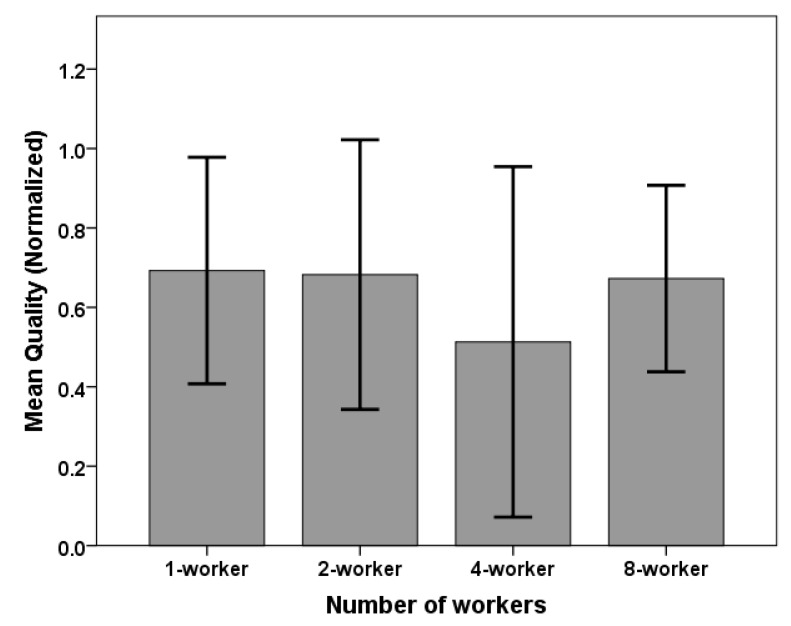
Dialogue quality: no significant difference in quality scores between all conditions.

We evaluated response appropriateness as a measure of the dialogue’s quality. Since each dialogue had a different length, we normalized the appropriateness scores by length (dividing quality scores by the dialogue’s length). We also ran a one-way ANOVA analysis to test for differences in the mean scores between all four conditions (Cf. Figure 6 and Table 5). We found no significant difference in quality scores between all four conditions: *F*(3,16) = 0.50, *p* = 0.687. Hence, we found that having more workers in queue does not significantly improve the dialogue’s quality.

### 6.4. Cost

The average cost of experiments was $4.93 (SD = 1.76) for one-worker, $6.60 (SD = 1.62) for two-worker, $8.59 (SD = 1.82) for four-worker, and $18.0 (SD = 4.02) for eight-worker conditions. We also conducted one-way ANOVA, results indicated that cost differed significantly: *F*(3,16) = 27.20, *p* < 0.0005. Post hoc Bonferroni comparisons indicated that this difference in cost was prominent between one- and eight-worker (*p* < 0.0005), two- and eight-worker (*p* < 0.0005) and four- and eight-worker (*p* < 0.0005) conditions. There was no difference between one, two, and four-worker conditions. The linear regression model explained 81.4% of the overall variance in the cost ($R^{2}$ = 0.814) based on the number of workers in the active queue which was significant (*F*(1,18) = 78.9, *p* < 0.0005, effect size (*d*) = 4.38). The linear equation was:

(5) Cost=2.54+1.87(number_of_workers)

### 6.5. Assessing the Quality of Robot Utterances Through Liwc

**Figure 7:**
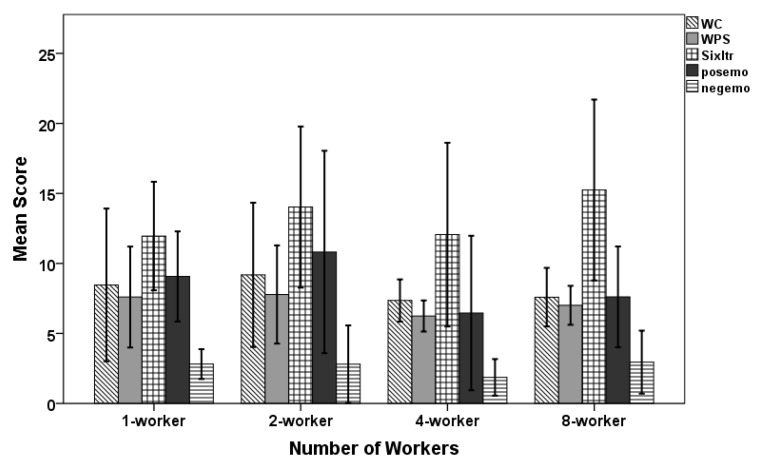
Using one-way ANOVA, we found no significant difference between mean scores of five categories across all conditions.

One-way analysis of variance (ANOVA) was conducted to find the difference in mean scores for five chosen variables across four conditions. We did not find any difference in the mean scores for WC (*F*(3,16) = 0.345, *p* = 0.793), WPS (*F*(3,16) = 0.524, *p* = 0.672), Sixltr (*F*(3,16) = 0.593, *p* = 0.629), posemo (*F*(3,16) = 1.032, *p* = 0.405) and negemo (*F*(3,16) = 0.496, *p* = 0.690)(cf. Table 6 and Figure 7).

**Figure 8:**
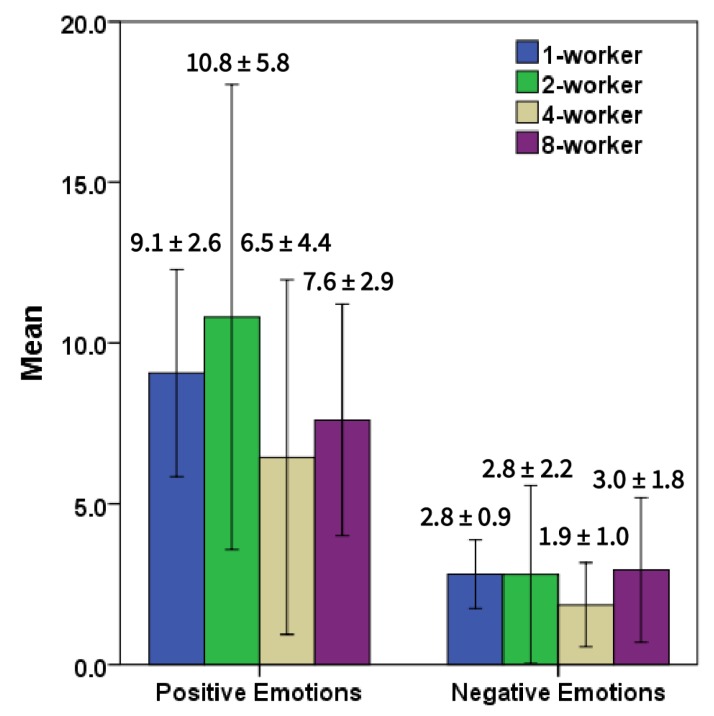
*t*-test revealed that words chosen by crowd workers contained more positive emotions than negative emotions across all conditions.

Across all conditions, it was revealed that the choice of words used by crowd workers while talking to an actress resulted in overall positive emotions. Figure 8 shows differences in scores between positive and negative emotions across all conditions. Using an independent *t*-test, we confirmed that this difference was significant between positive and negative emotions for one-worker (*t*(8) = 5.13, *p* = 0.001), two-worker (*t*(5.14) = 2.87, *p* = 0.034), four-worker (*t*(4.44) = 2.25, *p* = 0.05) and eight-worker (*t*(8) = 3.05, *p* = 0.016) conditions.

### 6.6. Average Waiting Time for Eliciting Multiple Responses

Future work can improve CoZ by combining crowd input with AI-generated input in a hybrid fashion. As there are multiple workers simultaneously controlling the speech of the robot, it is possible that more than one worker responds to the same user query. Therefore, an important question arises: How long would an algorithm need to wait before it receives enough responses to choose the best one from? To answer this question, we gathered all dialogue exchanges from the 2, 4 and 8-worker conditions. Here, corresponding to each user query we received more than one response on average from crowd workers in each of the conditions (cf. Table 7, column 3). Then we consider the time difference between the last robot utterance and the first robot utterance for the same user query. Finally, we calculated the mean scores for each condition (Table 7). To find the difference in mean scores between all three conditions, we conducted a one-way analysis of variance (ANOVA). Results indicated that there was no difference in mean scores: *F*(2,12) = 0.720, *p* = 0.507. Based on these findings, we conclude that the maximum time an algorithm should wait is around 6 s. During this duration, we can expect more than one crowd response.

### 6.7. Effect of Progress Bar and STT on Response Latency

To check whether a message was sent by a worker within the 9 s duration (maximum elapsing time of progress bar), we counted all such instances from our corpus (Table 8). A $\chi^{2}$ analysis (4 conditions x 2 (<9 s or above)) indicated that increasing the number of workers who engage with the conversational task would possibly increase the chance of messages to be composed and sent in less than 9 s, $\chi^{2}$ (3) = 26.65, *p* < 0.0005.

To check whether workers used the speech to text service while composing a response, we logged this information. There were three response cases for a worker: using speech to text (STT), simply typing a response (TYPED) or using both (MIXED), e.g., in case of STT failures, workers could edit the response. Out of 551 workers’ utterances, only 16 responses (2.9%) used speech to text, 13 used (2.36%) mixed while 522 (94.74%) responses were typed. This was surprising, since we had advised workers to use the STT in our instructions with an aim to yield a shorter response latency.

### 6.8. Experts’ Evaluation and Detailed Feedback

We hired two clinical psychologists to rate the quality of the dialogues on a seven-point Likert scale, where a score of 7 would indicate that the dialogue that transpired was highly professional. We found a strong Cronbach’s alpha value (α = 0.82) among our two raters. We also computed the Spearman’s rank–order correlation to determine the relationship between two ratings. We found a strong, positive correlation which was statistically significant ($r_{s}$(20) = 0.723, *p* < 0.001).

Next, we took the mean score of the two ratings given by our two experts and executed an ANOVA test to investigate differences in mean scores between the four conditions. We did not find any differences in the mean scores (*F*(3,16) = 0.123, *p* = 0.945). The average score was 3.9 (SD = 2.07) for one-worker, 3.7 (SD = 1.95) for two-worker, 3.3 (SD = 0.75) for four-worker, and 3.4 (SD = 1.91) for the eight-worker condition. The maximum score given by the first rater for each condition was 5, while the maximum score given by the second rater was 7 for one and two-worker condition, 4 for the four-worker condition and 6 for the eight-worker condition.

Given the fact that workers were not trained in clinical psychology and did not have prior experience in mitigating stress, these results are satisfactory and quite promising in terms of potential. We believe that with some training and implementing more sophisticated workflows, these results can be improved.

### 6.9. Qualitative Feedback from Psychologists

The psychologists provided the following comments regarding the performance of CoZ in powering this robot-human dialogue. We asked the professional psychologists to go through the whole dialogue and they chose the exemplary responses that we present below.

#### 6.9.1. Building upon User Strengths

CoZ tried to explore the user strengths and then built upon those to provide advice against stress: User: “I love cooking. Mostly I like cooking for other people. I don’t know many people yet”. Robot: “What is the last meal you cooked?” Asking her what the last meal she cooked reminds her that there is more to life than just academic studies and the stress that develops when studying. CoZ could build upon this ‘strength’ of the user (i.e., cooking) later on during the session by helping her to acknowledge her strengths. It is important, especially among people experiencing stress, to help them remember their own strengths. Especially when academics get difficult, people often internalize their struggle and begin to believe they are failures and generalize this belief to several areas of their life.

#### 6.9.2. Assessing Coping Skills

CoZ asked what the user has tried to do to overcome his anxiety and stress. An indicative excerpt: “What have you tried till now to overcome it?” In this way it assessed the user’s coping skills. In another indicative example, CoZ asked what does she like to do when not studying (e.g., “What do you like to do when not working on your research?”). This is also a desirable question during a coaching session to get the user thinking and talking about coping skills to manage stress. It is better to focus on one or two coping skills and make sure the user has a good grasp on how to apply it in their lives.

#### 6.9.3. Let the User Express Herself

CoZ let the user talk more and express herself before jumping to solutions. For instance, CoZ asked her questions about her experience in a new city (e.g., “Where did you move from? How long have you been in your new city?”). This is a great way to begin a session that allows the user a chance to express herself, her feelings, and her present causes of stress. It might seem like it will take too long and extend the session, but in reality, it is a time saver because allowing her to talk more and having CoZ reflect back and show empathy will help the user reveal the real root cause of her stress.

#### 6.9.4. Recalling Positive Things about Life

When it comes to people dealing with stress and anxiety, it is important to help them recall the factors in their lives that are positive and that they enjoy because often-times, stress and anxiety lead to and/or are fueled by negative/dysfunctional thoughts. For instance, CoZ asked: “What activity gives you the most joy in your life?”). Here CoZ is trying to guide the user out of that mindset and helping the user consider positive things about her life.

### 6.10. Suggestions for Improvements

Beyond the positive aspect of the crowd’s input, the professional psychologists had suggestions for further improving the quality of the coaching. We synthesized their suggestions and briefly report the most important ones.

#### 6.10.1. Coz Should Introduce Itself as a Coach

According to the feedback of the professional psychologists, a coach is neither a therapist or counselor nor a friend, but something in between like a ‘life guide’ or ‘mentor’ for the user. The psychologists suggested that it would be a good idea for the coach (here CoZ) to introduce itself as a life coach and (if possible) greet the user by her name. For example, saying something like this: “Hi John, I’m Pepper, your life coach. I’m ready to talk with you. What brings you to life coaching today?” According to the psychologists this would sound more professional and provide the user with confidence that she is doing more than just chatting with someone online.

#### 6.10.2. Reflect Back and Validate

Another suggestion was that it is important for a coach to reflect back and validate what the user is saying. For instance, instead of CoZ saying, “What are you stressed about?” it would be better here for CoZ to reflect back what the user is saying. For example, CoZ could say, “It sounds like your stress has gotten to the point that it’s starting to affect your life, even your studies. What in particular are you stressed about?”. Here, CoZ would show that not only is he listening to the user and is concerned about her, but is also validating the user’s stress. This will increase the user’s comfort in the session and help her to continue to open up.

#### 6.10.3. Avoid Unnecessary Small Talk

A final suggestion was that a coach should avoid unnecessary small talk. For instance, CoZ asked the user her age and whether she has a partner and the user immediately called her out when she stated, “ah personal question.” It is not appropriate for a life coach to ask such a question, especially asking whether she has ever had a partner in her life. Furthermore, during a session with Indian workers, CoZ mentioned prayer and went on to say to the user that God is always with her so she should “be happy”. Due its personal nature this feedback would not be appropriate for life coaching. Also, when mentioning God or religion, CoZ should first wait for the user to bring up this topic because CoZ does not know the user’s spiritual beliefs.

### 6.11. Discontinuities/Non-Cohesiveness in the Crowd Generated Conversations

In most of the dialogue exchanges, crowd workers maintained coherence and continuity in the conversation, nonetheless there were some cases where conversation diverged from the one topic to the new topic prematurely. There were various reasons that we will explore in this analysis. The purpose of presenting these findings is to show that such discontinuities are expected in real time crowd-powered dialogue systems and proper mechanisms should be devised to resolve them.

In order to find incoherence in conversations, we established some criteria after reviewing conversations in all four conditions. This includes abruptly switching a topic due to lack of experience, arrival of new workers, unnecessary small talk, discussion about the task, technical difficulties, and presence of spam workers who deviated the discussion. Based on this criteria, we counted all such instances from the dialogue. Table 9 shows total and mean discontinuities across all conditions.

#### 6.11.1. Switching Topics Prematurely

There were some dialogue exchanges where workers made an abrupt and odd transition without concluding the ongoing topic. One possible reason for deviating the conversation in an unnecessary way is the lack of experience of workers for this therapeutic task. An indicative excerpt of this form of disjunction is shown below. Here workers first propose short activities to relieve stress and then abruptly jumped to herbal remedies.

Robot: Doing short activities to get your mind off of your studies might help, and then you can come back with a clearer mind.

User: yeah, I can see how that’s work. What do you think of when you say short activities like what maybe?

Robot: Perhaps trying some herbal remedies

Workers can be trained to collaborate with fellow workers to communicate consistent messages unless a user deliberately switches to a new topic.

#### 6.11.2. Newly Joined Workers

Occasionally, when a new worker joined the session, he/she initiated the discussion with either greeting (’hello’, ’how are you?’) or requested a user to repeat her last utterance. This resulted in an odd transition and our actress had to restate her worries again. We observed this form of disjunction in the 4 and 8-worker conditions where a greater number of workers left and joined the session more frequently. This was surprising since we had shown the ongoing conversation to workers who were in the waiting state and they were well aware that they had to pick up the ongoing discussion. Furthermore, after they were redirected to the main conversational task, they could also see messages from other workers. In future work, simple automated methods can be employed to filter out such repeat requests or greetings.

#### 6.11.3. Small Talk

At times, workers engaged with the actress in unnecessary small talk which resulted in non-cohesive dialogues. The most common pattern that we observed was personal questions about the actress’ private life (’what is your age?’, or ’do you have any partner in your life?’). Workers can be easily trained to avoid asking personal questions to the user.

#### 6.11.4. Side Chatter

Sometimes, workers wrote their confusions about the task and instructions directly into the chat interface which resulted in an odd transition. Sample quotes of this form of disjunction are shown below:

Robot: Do you allow repeats? I feel like we didn’t finish our conversation properly.

Robot: where are the instructions?

#### 6.11.5. Technical Problems

Another form of disjunction occurred due to some technical issues experienced by workers during the task and as a result, they wrote their responses as complaints directly into the chat interface.

Robot: I have not heard voice if I can hear I will help to you.

Robot: video is OK but not have a sound

#### 6.11.6. Spam Worker

We had two spam workers that included out of context, worthless and rude responses. But when this happened, other trustworthy and serious workers took over and handled the conversation. Examples include:

Robot: who cares about your studies?

Another honest worker handled this situation by reflecting and validating the actress’ problem as follows:

Robot: You’re having trouble studying, but you can’t stop using your phone? I understand.

User: well I should have started with saying that I did get rid of my phone.

### 6.12. Performance of Pavilion

**Figure 9:**
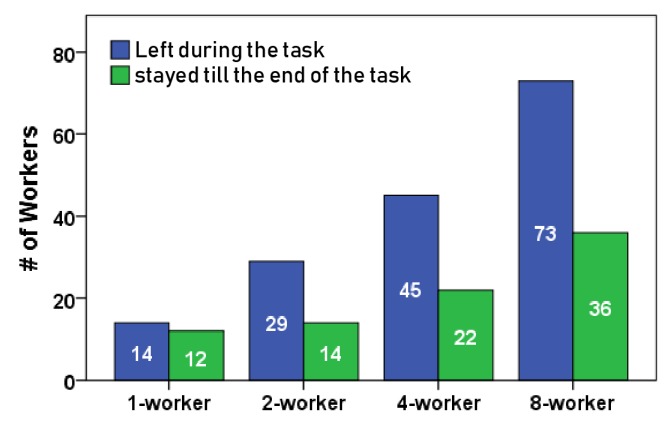
Number of workers who left during the conversational task vs. who stayed till the end of the task.

High departure rate: Figure 9 shows that more workers left during the task execution as compared to those who stayed till the end of the task. Since we were not sure about the exact length of a dialogue, we auto submitted all HITs from workers who stayed till the end of the task through admin control (workers were informed). We found a strong positive correlation between HITs that were submitted or returned during task execution and the ones that were submitted automatically at the end of the task (*r* = 0.98, *p* = 0.01). This further confirms the volatile nature of workers on MTurk and emphasizes how mechanisms for handling this asynchronous turnover of workers during a session is a key feature for enabling RTC.

Worker retention: Pavilion was able to retain the required number of workers in the active queue during the task execution (active workers at the start of session and at the end were the same) despite the high turnover rate of workers. When a worker left the active queue, Pavilion moved workers from the waiting to the active queue instantly or waited until a sufficient number of workers were available. We calculated the average time that Pavilion took to regain the required number of workers in the active queue when workers left during the task execution. The average was 0.07 s (SD = 0.04) for one-worker, 55 s (SD = 85.8) for two workers, 49.8 s (SD = 69.9) for four workers and 29.2 s (SD = 68.1) for eight workers. The reason it took longer for Pavilion to refill the active queue when more than one worker was needed was due to some workers leaving the task at the start of conversation and not having enough workers in the waiting queue to migrate straight away.

Recall requests and new HITs: In our experiments, a total of 123 recall requests were made to move workers from the waiting to the active queue for all conditions. Out of 123 recall requests, 47 were directly associated with reinstating the required number of workers in the active queue. To fulfill the deficiency of workers in waiting queue, Pavilion posted 24 new HITs during the study.

Active workers: Other than the first condition where exactly one worker was responsible for controlling the dialogue, we computed the average number of active workers in three conditions. We considered a worker as active if he/she contributed at least one message during the dialogue. We found that for one and two-worker conditions, the average number of active workers remained 1 and 2 respectively throughout the session. However, for four and eight-worker condition, the average was 3.2 (SD = 1.64) and 6.8 (SD = 2.39) respectively. This shows that apart from sustaining the target number of workers in the active queue, mechanisms are required to regularly prompt the idle workers or replace them with other workers.

## 7. Discussion

Our results indicate that increasing the number of workers in the queue did not improve the quality of the dialogue, but it did shorten the response latency. A linear regression analysis confirmed that adding a new worker in the queue can decrease the response latency by 0.6 s but at the expense of extra $1.87. Cost and latency remain inversely proportional to each other when we hire more workers while quality remains constant. Future research should explore the limits of this finding: namely the number of workers after which we observe diminishing returns for the extra investment. Our stringent qualification requirements on MTurk (98% approval rate and 1000 HITs submitted), can explain the results regarding the dialogue’s good quality. Costs can further be reduced by paying a fixed bonus for waiting tasks. For example, in a survey [50], a crowd worker suggested $0.50 for 10-min waiting time. In the imminent future, we will explore the impact of answer aggregation approaches on quality-related outcomes of CoZ.

### 7.1. Guidelines for Enabling Social Robotics to Handle Stress Management via RTC

#### 7.1.1. Handling Quality

(a) Training workers: The quality of the conversation can potentially be improved by training the crowd workers before they engage in the conversation. More specifically, for the case of managing stress that we studied in our experiment the psychologists suggested that the crowd workers could be advised to:

1. Allow the user to express his/her stressors/concerns without interruption (at least initially without interrupting or changing the topic).
2. Avoid simplistic answers/solutions that are also too general or not realistically applicable for the user (e.g., “just try to think positive” or “maybe you can just move to another apartment”).
3. Use empathy, understanding, and reflection to let the user know the robot is listening and cares about the users concerns.
4. Respond on topic and in line with the users expressed concerns/stressors.
5. Avoid jumping from topic to topic rather than maintaining an organization and structure to the session
6. Assess the coping skills that the user is currently employing/applying to try to manage stressors. This avoids giving advice about coping skills that the user may have already attempted.

We are currently exploring how can we structure the instructions given to workers based on Motivational interviewing (MI) [53]. MI is very useful tool which could enhance the performance of non-experts from a crowdsourcing platform to provide reasonable coaching to people who are stressed. MI is defined as [53]: “a collaborative conversation style for strengthening a person’s own motivation and commitment to change”. It includes four processes: engaging, focusing, evoking and planning. In addition to that, we can add reflective listening based on the psychologists’ feedback. Reflective listening is very useful tool to understand the inner struggle and commiserate with the stressed person.

(b) Automated response selection: An important measure to improve the quality of responses is to elicit multiple responses from crowd workers in a short span of time and then to use an automatic method to select the best response. In our study the average time it took to elicit multiple responses was about 6 s. One way to ensure quality is to select the best response provided by the crowd within at least such a time span. Nevertheless, this is a complex NLP problem which requires further investigation. For instance, given more than one response to a single user utterance, one can select the one that is most appropriate.

(c) Structuring the dialogue: Cross-checking the responses generated by the crowd, we found that workers in the four- and eight- workers conditions (who joined the session later) said ‘hello’ in the middle of a conversation which resulted in re-starting the conversation. The system could support workers in structuring the dialogue by providing some suggestions of the conversation structure and context, e.g., whether currently the dialogue is about greeting, exploring (asking questions) suggesting and proposing solutions, or closing. Also, workers could be made explicitly aware of the robot’s attributes (e.g., its name) and its environment (e.g., weather, time, location) to avoid situations such as (indicative excerpt): Robot: Yeah, it is time for breakfast—User: Time for breakfast? Well it’s one o’clock in the afternoon here.

#### 7.1.2. Handling Latency:

There is an important question that remains: How could the system handle a failure of the crowd to produce a response within an acceptable delay? The most effective approach for reducing the latency involves combining AI with human computation techniques. In social robotics, it is known that if a robot takes more than two seconds to respond then it can induce frustration in users [54]. One solution that has been developed to support artificial conversational agents is to ‘buy time’ with conversational fillers or acknowledgement tokens [55]. These conversational fillers can be simple (e.g., “well” and “uh”) or complex (e.g., “that is an interesting question, let me think about it”). This way, the crowd would still be handling the essence of the dialogue while CoZ would aim to yield a more positive user experience and a better perception of system latency.

Another way to reduce the latency can be to support workers with auto-completion; when a worker starts typing a message, CoZ could propose a list of suitable replies derived from predefined messages from a corpus of dialogues. The role of workers would then be to select the responses that they think are more appropriate or modify them to generate new ones. Given the fact that workers preferred to compose messages by typing instead of using speech to text, such interventions would allow them to swiftly compose and send messages.

#### 7.1.3. Handling Privacy

If one would want to deploy a social robot operated by anonymous crowd-workers in a realistic scenario, then one needs to address privacy issues. A prior study that favors our crowd-based approach showed that participants felt more trust for the robot which was controlled by multiple teleoperators than a robot controlled by a single operator [56]. One possible reason could be that users interacting with the robot might treat an anonymous crowd as less threatening than teleoperators who are collocated and known to experimenters. To protect the privacy of end-users, different filtering techniques can be used. For example, color-skewed super-pixel filter [26] was considered acceptable for preserving privacy for end-users while obscured view filtering method was considered good for recognizing objects comfortably for teleoperators. Furthermore, we recommend that end-users should be given full control to switch between a privacy-preserving and a “normal” mode. For example, when teleoperators need fine-grained information to execute a task, they can request users, by using Pepper’s tablet, to grant them permission to temporarily suspend privacy veils.

### 7.2. Potential for Re-Purposing

From a technical perspective, developers can integrate CoZ by other robots than Pepper. For example, no changes in the implementation are needed to use CoZ for the NAO robot (https://www.softbankrobotics.com/emea/en/nao). Since we have chosen a modular approach to develop CoZ, one can replace an OOCSI script which communicates with the Pepper robot to any other robot by writing a script using relevant API commands. No other changes are needed in the rest of the application.

CoZ can enable researchers to run experiments to collect conversational dialogue data that can be used to train a fully automated dialogue management system in different contexts (e.g., chitchat, mental health). This method is quite affordable and scalable than traditional Wizard of Oz experiments.

## 8. Future Work

For our next steps, we are planning to replace the actress and see how this crowdsourcing technique (with coaching and instructions for workers) works in the wild. We have planned two studies after training workers based on the guidelines presented in Section 7.1.1 and employing conversational fillers to buy some time when workers are busy in typing their responses.

First, we plan to deploy our CoZ powered robot to our department for students who are feeling stress due to study burden and problems with their private life. Since university students are highly vulnerable population, there is also an opportunity to investigate about the effect of media (audio and video) on the quality of conversations, engagement (time spent with the robot), disclosure of sensitive information and privacy aspects. For this purpose, we plan to conduct a between-subjects user study, wherein the robot broadcasts both audio and video to crowd workers in one condition, as opposed to broadcasting only the participants’ audio cues in the other condition. If the study shows no significant difference between quality of conversations, engagement and privacy aspects, then perhaps we do not need to broadcast video of the users to the anonymous workers.

Second, we plan to deploy CoZ in an elderly care house to act as a companion for older adults to treat their loneliness and stress. A prior study [57] shows that older adults felt more concerned about their privacy-enhancing behaviours when they were monitored by a camera (67%) as compared to an anthropomorphic robot (17%). Hence, this study will help us to focus more on the quality of interaction and crowd-based life-coaching than addressing privacy issues.

To further reduce the risks in these interactions, especially for a vulnerable population, we will include the supervision of trained staff to carefully evaluate the technique and intervene whenever necessary to make sure that there is no risk in these interactions. We can also train workers to avoid requesting personal information and we can employ automated methods to filter out queries containing sensitive information. In addition, computer vision techniques such as silhouetting or avatars and altering the voice of the person using audio modulation techniques can be used to hide the identity of the person talking to the robot.

## 9. Conclusions

We introduce Crowd-of-Oz (CoZ)—a system designed to enable real time crowdsourcing for teleoperating a social robot in complex social conversation. CoZ extends earlier works in crowdsourcing and human–robot interaction where the response time of a couple of seconds is desirable, in situations where crowd-workers have to apply human intelligence skills exceeding the current state of the art in AI. In this study, we leveraged social communication skills of crowd workers in MTurk to provide meaningful answers that take into account the rich contextual information for coaching someone with stress. Our experimental evaluation of CoZ systematically examined how the number of workers affects the quality, cost and latency of CoZ. Our results indicate that increasing the number of workers did not improve the quality of the dialogue, but did shorten the response latency at the expense of cost. Regarding the effectiveness of dialogues, the feedback from professional clinical psychologists shows that crowd workers recruited in real-time provided a satisfactory coaching performance. Opportunities to further improve CoZ’s performance include: creating a hybrid system combining both AI and crowd wisdom to (a) reduce latency by employing conversational fillers and auto-completion features; (b) improve dialogue quality with new workflows and UIs to improve the structure of the dialogue and enriching context awareness of workers; (c) creating new workflows for training workers as per recommendations received from professional psychologists.

## Figures and Tables

**Table 1:** Summary of the key differences between Crowd of Oz (CoZ) and other crowd-powered conversational agents.

Reference	Use Case	Input	Output	Device
Chorus [41]	Information retrieval	User queries in natural language	Text message	Mobile phones or PC
Evorus [42]	Information retrieval	User queries in natural language	Text message	Mobile phones or PC
VizWiz [40]	Assisting blind users to interact with devices	Video stream and recorded audio question + text	Audio through voice-over screen reader	Mobile phones
Chorus:view [43]	Assisting blind users to interact with devices	Video stream and recorded audio question + text	Audio through voice-over screen reader	Mobile phones
CrowdBoard [45]	Creativity	Write ideas on sticky notes	Textual ideas from crowd workers	Digital whiteboard
InstructableCrowd [44]	Programming	User’s problem in natural language	IF-THEN rules	Mobile phones
CoZ	Live conversational task for stress management	Real time audio and video feed + transcribed text messages	Animated Speech by Pepper robot and text message displayed on the Pepper robot’s Tablet	Pepper or NAO robot

**Table 2:** Summary of the key differences between CoZ and other systems from crowd or web robotics.

Reference	Use Case	Input	Output	Robot
Legion [20]	Robot navigation	Video stream of rovio robot + arrow key presses	Robot movement	Rovio robot
CrowdDrone [46]	Drone navigation	Simulated or real imagery from drone’s camera + arrow key presses	Robot movement	Drone robot
EURECA [21]	Scene manipulation	Natural language query + scene manipulation (zoom, pan, orbit) + selection tools	Segmented and labelled objects	Fetch robot
Robot Management System (RMS) [47]	Robot navigation + manipulation	Arrow keys for changing direction + camera feeds + 2D map + slider control to alter speed + arm controls	Robot movement and object retrieval	PR2 robot
Learning from demonstration [48]	Robot learning	Web interface for controlling a robot	Robot movement	iRobot
CoZ	Live conversational task for stress management	Real time audio and video feed + transcribed text messages	Animated speech by Pepper robot and text message displayed on the Pepper robot’s Tablet	Pepper or NAO robot

**Table 3:** Problems or situations mentioned by an actress during sessions and examples of corresponding crowd responses.

Problem/Topic	Indicative Excerpt
Opening a discussion	User: I have been stressed for the past few weeks really and it’s very difficult to focus on my studies and it would be nice just to talk about that. Robot: What is stressing you about your studies?
Moving to a new city	User: I moved from Belgium to the Netherlands 3 months ago to do this minor so its new city and new house I am living in. Robot: Are there any language barriers you have to deal with? User: Not really because in Belgium we speak Flounderish and in Holland, they speak Dutch and it’s the same language. Robot: So, the differences between your studies and studying life are feeling overwhelming?
Difficulty in study	User: So, I’m doing a minor here in Eindhoven and it’s new subjects and it’s so different working than before…I am anxious about it⋯ it’s a new style of working…new content, so I do not know how to deal with that. Robot: Why did you chose this subject? User: I thought it was interesting… it is more focused on user experience. Robot: But learning it is not giving you happiness? User: I think it will give me… we disagree maybe because I think my study gives me fulfillment. Robot: How come fulfilment and stress at same time?
Difficulty in finding people for an interview	User: I need to find people to interview and I don’t know so many people. So, I have tried putting an Ad up, but it didn’t work. It mixes up with all my planning. Robot: Try social media for getting connections. User: so, what would you put up on social media who would like to go out for a coffee with me then. Robot: Yes, they have groups for locals on places like Meetups.
Noisy roommates	User: I was hoping I would get some sleep, but I have 5 extremely noisy roommates. Robot: Can you try playing low volume rain sounds or something to drown them out? User: What is white noise? Robot: A background sound that’s relaxing to listen to that will drown out the loud sounds that your roommates make.
Showing empathy	User: I am saying that I am very stressed and that I like to speak about that, and I can’t focus on my studies Robot: Oh, that’s terrible what can I do to help?

**Table 4:** Different solutions provided by crowd workers to allivate the stressful thoughts of an actress.

Solutions	Indicative Excerpt
Mindfulness	Robot: I just do mindfulness, where I’m no judgmentally aware of my thoughts and feelings
Prayers	Robot: Do prayer Robot: God is always with u
Exercise	Robot: Walking is good to clear your mind.
Having a dog	Robot: maybe get a dog Robot: Do you like dogs?
Socializing	Robot: Sometimes talking to your family helps too. They can give you advice and love Robot: Maybe you could do some volunteer work in the area to try to meet some new people
Playing sports	Robot: you can try playing sports as well
Sleeping aid	Robot: I’d definitely say sleep will help you a lot. You’ll be able to focus a lot more. Maybe try taking a nap during the day if you can find a time when your roommates are gone.
Taking a break from work	Robot: Perhaps clearing some free time in your schedule to relax while going through this transition would help.
Voicing one’s thoughts aloud	Robot: voice your thoughts out loud.
Focusing on one thing	Robot: It sounds like you need to break down your bigger problems into smaller parts to begin with.
Using calming teas	Robot: I drink hot tea and think about my past. Robot: take some calming teas.
Using ear plug or white noise machine	Robot: Have you tried ear plugs or a white noise machine?
Watching something interesting	Robot: watch something that you find funny or interesting Robot: watch YouTube
Listen to music	Robot: Another thing you could do is try and listen to music when stressed it is a great way to relax.
Yoga	Robot: Have you tried exercise or yoga?
Miscellaneous	Robot: Short walks or exercise, writing, meditation, watching a TV show, or talking to a friend.

**Table 5:** Mean latency and quality scores for all conditions.

Condition	Latency		Quality	
	Mean	SD	Mean	Mean
1-worker	8.82	2.95	0.69	0.23
2-worker	6.79	2.25	0.68	0.27
4-worker	6.79	1.96	0.51	0.35
8-worker	4.12	0.40	0.67	0.19

**Table 6:** Mean scores for five chosen categories calculated through linguistic inquiry and word count (LIWC) tool.

Condition	WC	WPS	Sixltr	Posemo	Negemo
1-worker	8.5±4.4	7.6±2.9	12.0±3.1	9.1±2.6	2.8±0.9
2-worker	9.2±4.2	7.8±2.8	14.0±4.6	10.8±5.8	2.8±2.2
4-worker	7.3±1.2	6.2±0.9	12.1±5.3	6.5±4.4	1.9±1.1
8-worker	7.6±1.7	7.0±1.1	15.2±5.2	7.6±2.9	3.0±1.8

**Table 7:** Average waiting time for eliciting multiple responses (first column), avg. responses per user query and maximum responses per condition (Max).

Condition	Avg. Waiting Time	Avg. Responses/User Query	Max.
2-worker	6.88 ± 2.81	1.25 ± 0.17	4
4-worker	6.60 ± 1.29	1.40 ± 0.23	6
8-worker	5.56 ± 0.71	1.53 ± 0.20	5

**Table 8:** Number of messages sent before and after 9 s.

Condition	Number of Workers			
	1	2	4	8
<9 s	65	88	79	130
>9 s	38	31	30	14

**Table 9:** Summary of discontinuities in the crowd generated conversations.

Condition	Total	Mean (SD)
1-worker	11	2.2 (2.9)
2-worker	15	3.0 (2.9)
4-worker	28	5.6 (4.3)
8-worker	36	7.2 (4.8)

## Abbreviations

The following abbreviations are used in this manuscript:

CoZ	Crowd of Oz
MTurk	Amazon Mechanical Turk
HIT	Human intelligence task
AI	Artificial intelligence
RTC	Real time crowdsourcing
AV	Audio–video
STT	Speech to text
MI	Motivational interviewing
LIWC	Linguistic inquiry and word count
WC	Word count
WPS	Words per sentences
Sixltr	Six letter
posemo	Positive emotion
negemo	Negative emotion
coproc	Cognitive process
percept	Perceptual
